# Ca^2+^ and force during dynamic contractions in mouse intact skeletal muscle fibers

**DOI:** 10.1038/s41598-023-51100-5

**Published:** 2024-01-06

**Authors:** Atsuki Fukutani, Håkan Westerblad, Kent Jardemark, Joseph Bruton

**Affiliations:** 1 Faculty of Sport and Health Science, Ritsumeikan University, 1-1-1 Noji-higashi, Kusatsu, Shiga 525-8577 Japan; 2 Department of Physiology and Pharmacology, Karolinska Institute, Solna, Sweden

**Keywords:** Cell biology, Physiology

## Abstract

Muscle fiber force production is determined by the excitation frequency of motor nerves, which induce transient increases in cytoplasmic free Ca^2+^ concentration ([Ca^2+^]_i_) and the force-generating capacity of the actomyosin cross-bridges. Previous studies suggest that, in addition to altered cross-bridge properties, force changes during dynamic (concentric or eccentric) contraction might be affected by Ca^2+^-dependent components. Here we investigated this by measuring [Ca^2+^]_i_ and force in mouse muscle fibers undergoing isometric, concentric, and eccentric contractions. Intact single muscle fibers were dissected from the flexor digitorum brevis muscle of mice. Fibers were electrically activated isometrically at 30–100 Hz and after reaching the isometric force plateau, they were actively shortened or stretched. We calculated the ratio (relative changes) in force and [Ca^2+^]_i_ attained in submaximal (30 Hz) and near-maximal (100 Hz) contractions under isometric or dynamic conditions. Tetanic [Ca^2+^]_i_ was similar during isometric, concentric and eccentric phases of contraction at given stimulation frequencies while the forces were clearly different depending on the contraction types. The 30/100 Hz force ratio was significantly lower in the concentric (44.1 ± 20.3%) than in the isometric (50.3 ± 20.4%) condition (*p* = 0.005), whereas this ratio did not differ between eccentric and isometric conditions (*p* = 0.186). We conclude that the larger force decrease by decreasing the stimulation frequency during concentric than during isometric contraction is caused by decreased myofibrillar Ca^2+^ sensitivity, not by the decreased [Ca^2+^]_i_.

## Introduction

Muscle contraction is regulated by Ca^2+^ released from the sarcoplasmic reticulum in response to sarcolemmal depolarization^1^. The released Ca^2+^ binds to contraction-regulating sites on troponin C, which permits tropomyosin to move, and consequently, myosin heads can bind to uncovered actin sites and thereby form force-generating cross-bridges^2^. When the frequency of motor nerve-induced depolarizations of the sarcolemma increases, the free cytosolic Ca^2+^ concentration ([Ca^2+^]_i_) increases, allowing muscle cells to contract with increasing force.

Many studies have examined the force-frequency relationship in human muscles in vivo^3,4^, in isolated whole mammalian muscles^5–7^, and isolated single intact fibers^8,9^, and consistently reported that about 100 Hz is needed to induce the isometric maximal force. However, few studies have investigated the force-frequency relationship in dynamic contractions such as concentric or eccentric contractions. de Haan et al.^10^ examined the force production of the rat medial gastrocnemius in situ during isometric or concentric contractions and found that the force-frequency relationship differed between isometric and concentric contractions, i.e., a higher stimulation frequency was needed to induce the maximal force in concentric contractions. Similarly, in the human quadriceps, a higher stimulation frequency was needed to achieve the maximal joint torque in concentric than in isometric contractions^11^. Thus, these studies imply that the force-frequency relationship differs between isometric and concentric contractions. To explain this phenomenon, two possibilities have been considered. First, the amount of Ca^2+^ released by a given stimulation frequency decreases when a muscle shortens. Second, a higher [Ca^2+^]_i_ is needed in concentric than in isometric contractions. To clarify which possibilities (or both possibilities) is true, [Ca^2+^]_i_ measurement is needed. In addition, this relationship has not been examined in eccentric contraction.

Therefore, the purpose of this study was to examine the [Ca^2+^]_i_- and force-frequency relationships by measuring the [Ca^2+^]_i_ during isometric and dynamic contractions in intact mouse single flexor digitorum brevis (FDB) fibers. We hypothesized that [Ca^2+^]_i_ is not different irrespective of contraction types when the stimulation frequency is the same, and consequently, differences in the force-frequency relationship is explained by different Ca^2+^ sensitivity.

## Materials and methods

### Sample preparation

Experiments complied with the Swedish Animal Welfare Act, the Swedish Welfare Ordinance, and all applicable regulations and recommendations from Swedish authorities. All methods reported in this manuscript are in accordance with ARRIVE guidelines^12^. The study was approved by the Stockholm North Ethical Committee on Animal Experiments (N120/13). Mice (C57/bl6) were housed in a temperature-controlled environment with a 12-h light–dark cycle and had free access to standard rodent chow and water. Mice were euthanized by rapid cervical disarticulation and the FDB muscles were removed from the hind limb.

### Experimental settings

Single muscle fibers were isolated from FDB muscles by mechanical dissection in a custom-made dissection trough under a stereomicroscope using fine forceps and micro-iris scissors^13^. Once a single fiber was isolated, platinum T-shaped clips were placed on the two tendons. FDB fibers were incubated in the membrane-permeable fluorescent Ca^2+^ indicator indo-1 AM (7.7 µM, Thermo Fisher Scientific, Stockholm, Sweden) for 60 min and washed for a further 30 min. Next, the fiber was mounted in a perfusion chamber (801C, Aurora Scientific Inc. Canada) by attaching one of the T-shaped clips to a force transducer (403A, Aurora Scientific Inc. Canada) and the other T-shaped clip to a length controller (315C, Aurora Scientific Inc. Canada). The chamber was placed on an inverted microscope (Diaphot 200, Nikon, Japan). Fibers were superfused with Tyrode solution (in mM): NaCl, 121,KCl, 5.0; CaCl_2_, 1.8; MgCl_2_, 0.5; NaH_2_PO_4_, 0.4; NaHCO_3_, 24.0; EDTA, 0.1; glucose, 5.5 and 0.2% fetal calf serum. The solution was continuously bubbled with 95% O_2_ and 5% CO_2_ to keep the bath pH at 7.4. All experiments were performed at room temperature (24–26 °C). Fibers were electrically stimulated (Pulsemaster A300, World Precision Instruments, UK) through platinum plates placed parallel to the long axis of the fiber. Before the start of each experiment, the length of each FDB fiber was adjusted using 70 Hz, 350 ms tetani at 1–2 min intervals to the length at which the maximal isometric tetanic force was produced. All subsequent contractions were commenced at this optimal length.

### Ca^2+^ and force measurements during contractions

**Figure 1:**
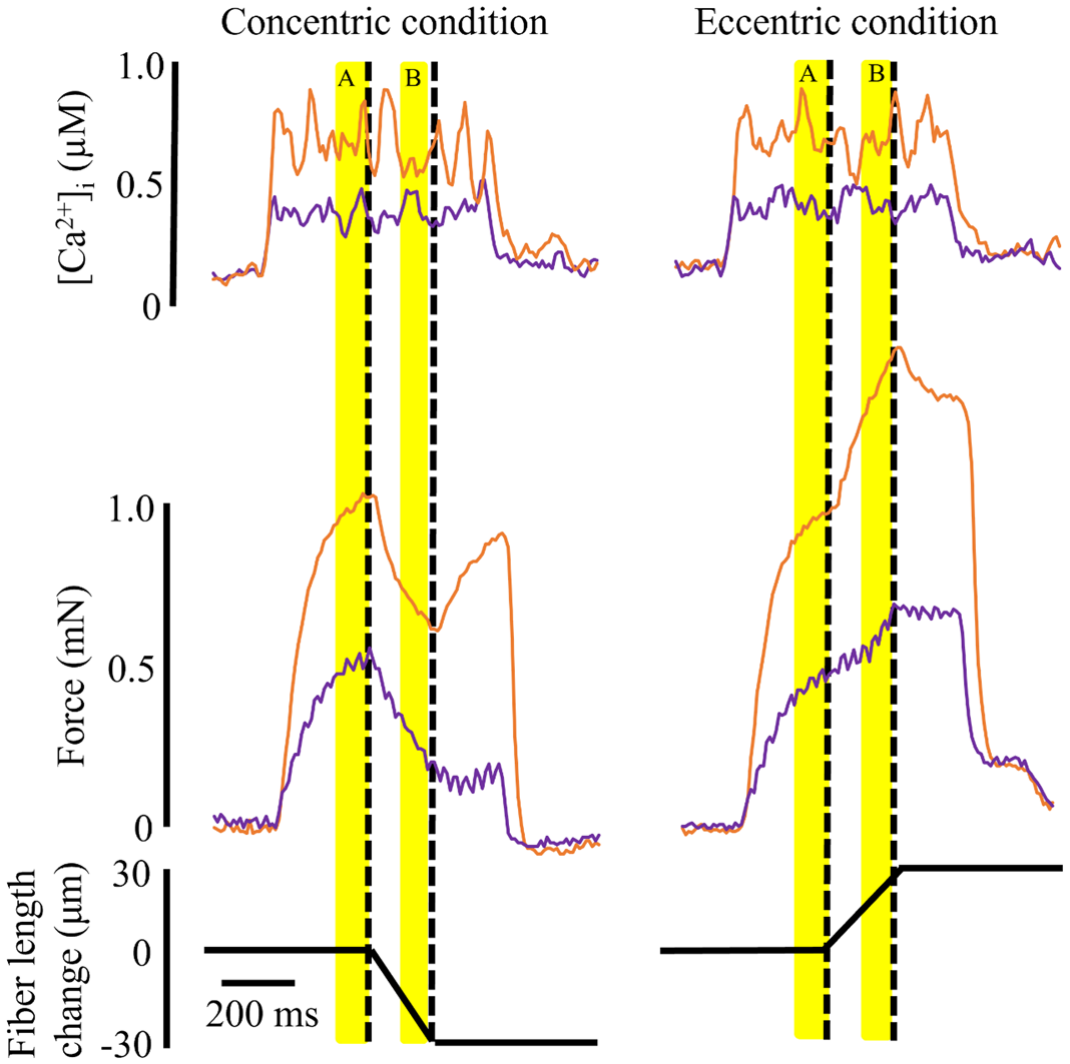
A representative sample of [Ca^2+^]_i_, force, and fiber length changes attained in the concentric (left panel) and eccentric (right panel) conditions. Orange traces, 100 Hz stimulation; purple traces, 30 Hz stimulation. The length changes are indicated by the thick dashed black lines. Yellow shading indicates the isometric (**A**) and concentric/eccentric (**B**) measurement phases (100 ms duration).

Fibers were stimulated to contract for a total of 700 ms. Typical records of [Ca^2+^]_i_, force, and length changes are shown in Fig. 1. In order to measure [Ca^2+^]_i_ and force under isometric and concentric or eccentric conditions in the same contraction, the initial 300 ms of each contraction was always isometric. Thereafter, fibers were actively shortened or lengthened by 30 μm (about 5% of the fiber length) over 200 ms, and 200 ms after the end of this length change, stimulation was ended. We used a low speed of shortening/lengthening and small changes in length to only induce modest changes in force when going from isometric to concentric/eccentric contraction (~ 30% decrease in concentric and ~ 60% increase in eccentric contractions), which facilitates assessments of changes in force-frequency and force-Ca^2+^ relationships. Stimulation frequencies of 30, 40, 50, 60, 70, 80, 90, and 100 Hz were used in this study. Successive contractions were separated by at least one minute of rest. Before and after the concentric and eccentric trials, the maximal isometric force was checked to determine if the fiber had been damaged, and if maximal tetani force had decreased by more than 10% all data from that fiber were discarded.

We used the indo-1 to measure [Ca^2+^]_i_ because this ratiometric indicator is little affected by fiber movement during contractions^14^. Moreover, our study is focused on the relation between changes in force and [Ca^2+^]_i_ during contractions. Therefore, we consider it advantageous to use indo-1 with kinetics (Ca^2+^ off-rate in muscle fibers of ~ 50 s^–1^) similar to those of the contraction-regulatory Ca^2+^-binding sites of troponin C (Ca^2+^ off-rate 20 s^−1^), and that this outweighs the fact that indo-1 is relatively slow and cannot readily report the peak amplitude and time-course of individual action potential-triggered [Ca^2+^]_i_ transients^15^. Indo-1 was excited at 360 ± 5 nm and the emitted fluorescent light was recorded at 405 ± 5 nm and 495 ± 5 nm using an imaging system consisting of a xenon lamp, a monochromator, and two photomultiplier tubes (Horiba, Wedel, Germany). The ratio of the indo-1 emissions at 405 and 495 were converted to [Ca^2+^]_i_ as described previously^16^.

Force data were recorded at 1000 Hz and indo-1 fluorescence at 100 Hz, and both were stored on a computer for later analysis with dedicated software (Aurora Scientific Inc. Canada for force records and FeliX32 Analysis, Horiba, Wedel, Germany for the indo-1 records). For both concentric and eccentric conditions, the isometric indo-1 ratio and force were measured as the mean values obtained during the 100 ms before the onset of the length change (Fig. 1, zone A). The concentric or eccentric indo-1 ratio and force were measured as the mean value during the 100 ms before the end of the length change (Fig. 1, zone B). To simplify comparisons of the [Ca^2+^]_i_- and force-frequency relationships in isometric, concentric, and eccentric contractions, we focused on comparisons between measurements of contractions at 30 Hz, which produces forces on the steep part of the force-frequency relationship, and at 100 Hz, which gives near-maximal forces.

### Statistics

Force and [Ca^2+^]_i_ at given stimulation frequencies in the concentric condition (isometric versus concentric) and in the eccentric condition (isometric versus eccentric) were compared using a repeated measure two-way ANOVA (frequency and contraction type). Differences between [Ca^2+^]_i_ under isometric and concentric or eccentric conditions in 100 and 30 Hz contractions were assessed using paired t-test. Differences in 30/100 Hz [Ca^2+^]_i_ and force ratios between isometric and concentric or eccentric conditions were tested with paired t-test. Statistical analyses were performed using SPSS (version 20, IBM, Tokyo, Japan), with the level of significance set at *p* < 0.05. All data are shown as mean and standard deviation (SD) or as mean and individual values when mentioned.

## Results

**Figure 2:**
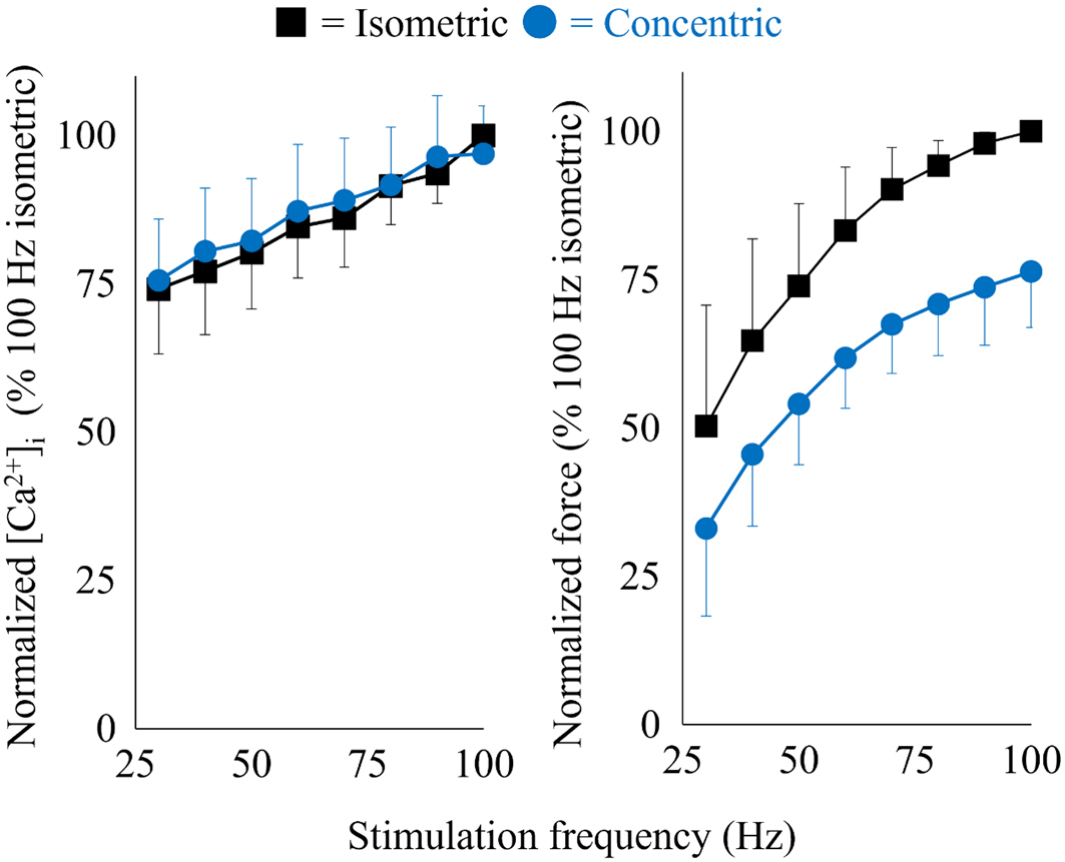
[Ca^2+^]_i_ -frequency relationship (left panel) and force-frequency relationship (right panel) measured in the isometric phase and the following concentric phase of shortening contractions. Values are mean ± SD of 8 fibers. Values obtained during the isometric phase of 100 Hz contractions were set to 100% in each fiber. Note that the error bar for isometric normalized force at 90 Hz was smaller than the symbol.

**Figure 3:**
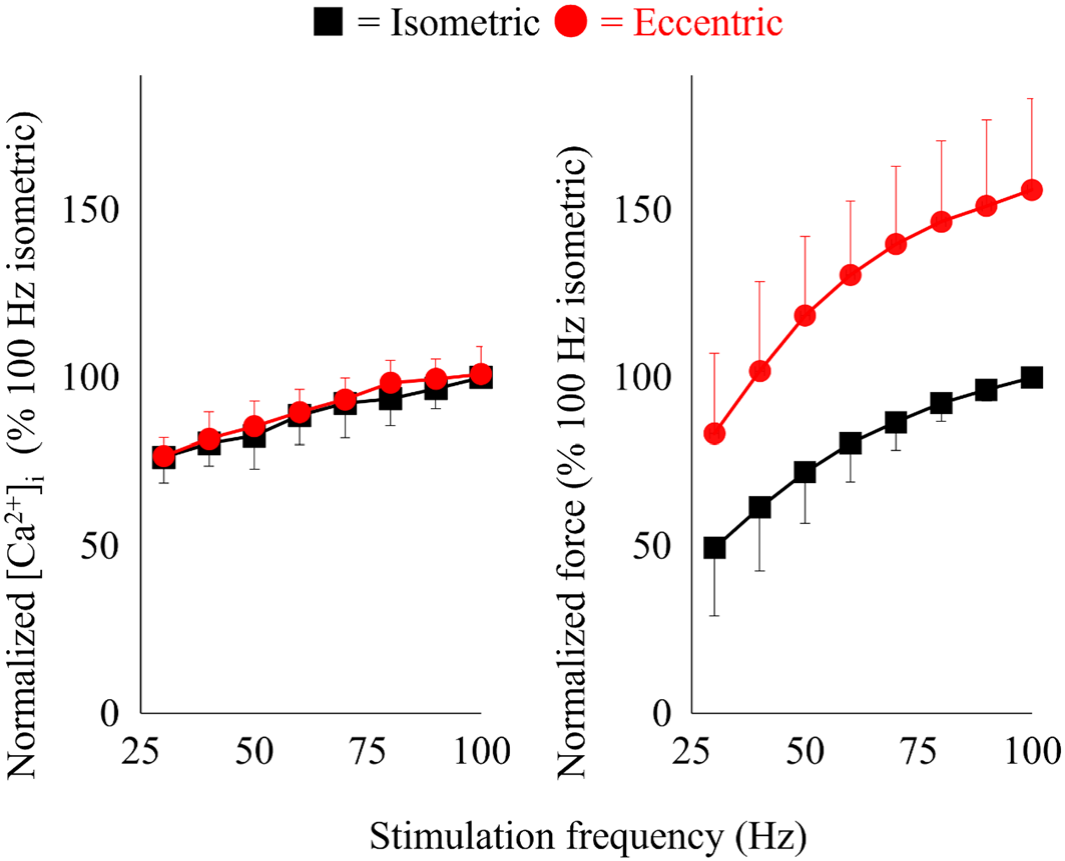
[Ca^2+^]_i_ -frequency relationship (left panel) and force-frequency relationship (right panel) measured in the isometric phase and the following eccentric phase of stretching contractions. Values are mean ± SD of 8 fibers. Values obtained during the isometric phase of 100 Hz contractions were set to 100% in each fiber. Note that the error bar for isometric normalized force at 90 Hz was smaller than the symbol.

**Figure 4:**
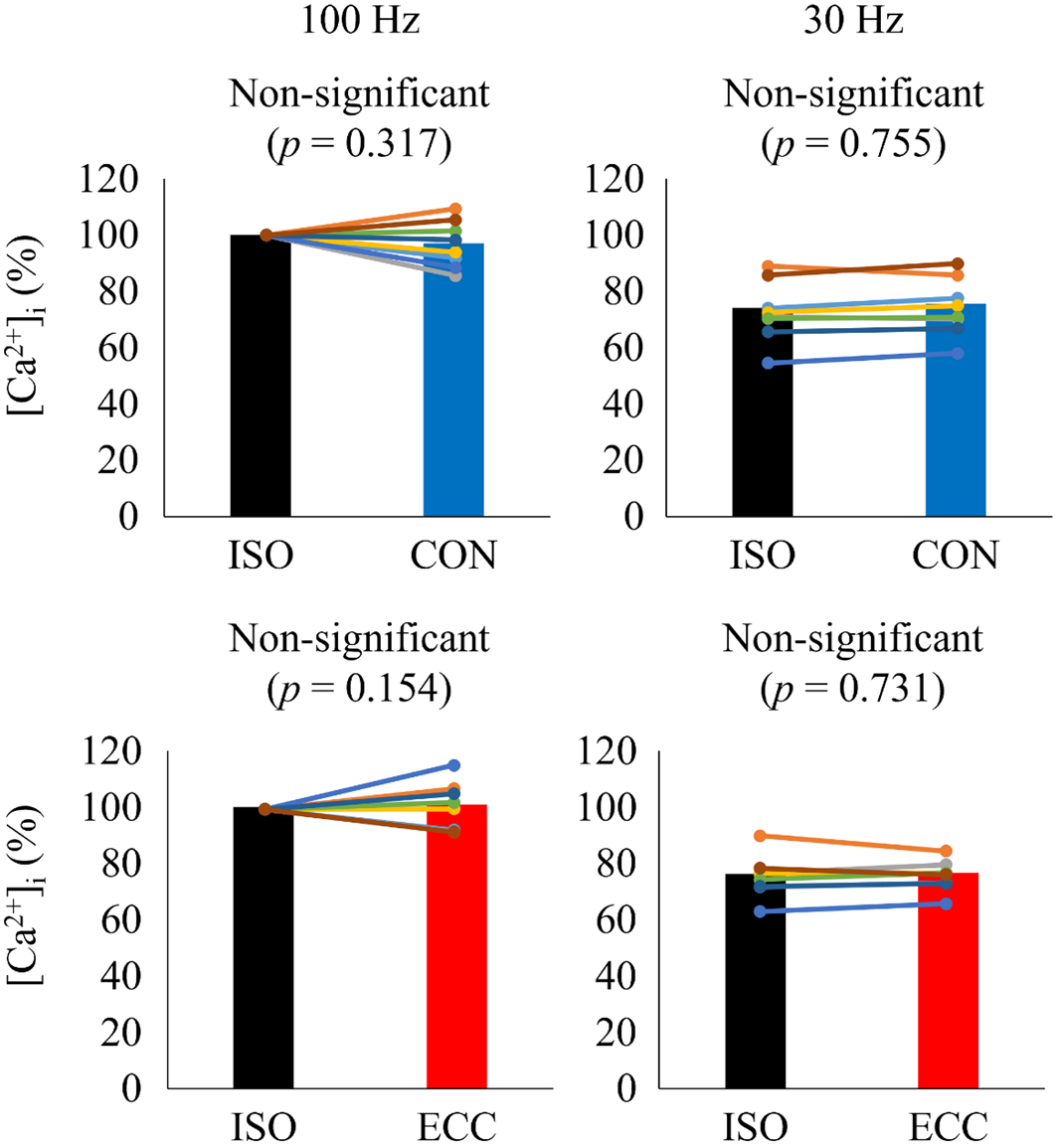
Normalized [Ca^2+^]_i_ during the 100 Hz (left) and 30 Hz tetani (right) attained in the concentric condition (upper panels) and eccentric condition (lower panels). Values obtained during the isometric phase of 100 Hz contractions were set to 100% in each fiber. Bars show mean values and each plot connected with a line shows individual values from 8 fibers. ISO, isometric phase (measured during yellow shading A in Fig. 1); CON/ECC, concentric/eccentric phase (measured during yellow shading B in Fig. 1).

Mean plateau tetanic [Ca^2+^]_i_ and force attained by the 100 Hz stimulation performed at the start of the experiment (0.695 ± 0.321 µM and 0.92 ± 0.58 mN) were similar to those performed at the end of the experiment (0.666 ± 0.337 µM and 0.94 ± 0.59 mN). The [Ca^2+^]_i_ and force during the isometric phase did not differ between contractions with shortening and lengthening (*p* = 0.278 for the [Ca^2+^]_i_ and *p* = 0.611 for the force). Figures 2 and 3 show the normalized [Ca^2+^]_i_-frequency and force-frequency relationships during the isometric and dynamic parts of tetanic contractions. It is clear that there was no significant difference in the tetanic [Ca^2+^]_i_ recorded during the isometric phase or dynamic phase of contractions (left panels in Figs. 2 and 3). Tetanic force clearly differs between the isometric and dynamic contraction phases (no significant interaction; *p* = 0.937) with force being significantly lower during the concentric and higher during the eccentric phase (*p* < 0.001; right panels in Figs. 2 and 3). Figure 4 shows that normalized tetanic [Ca^2+^]_i_ values attained in 100 Hz and 30 Hz contractions were not significantly different between isometric and concentric or eccentric conditions.

**Figure 5:**
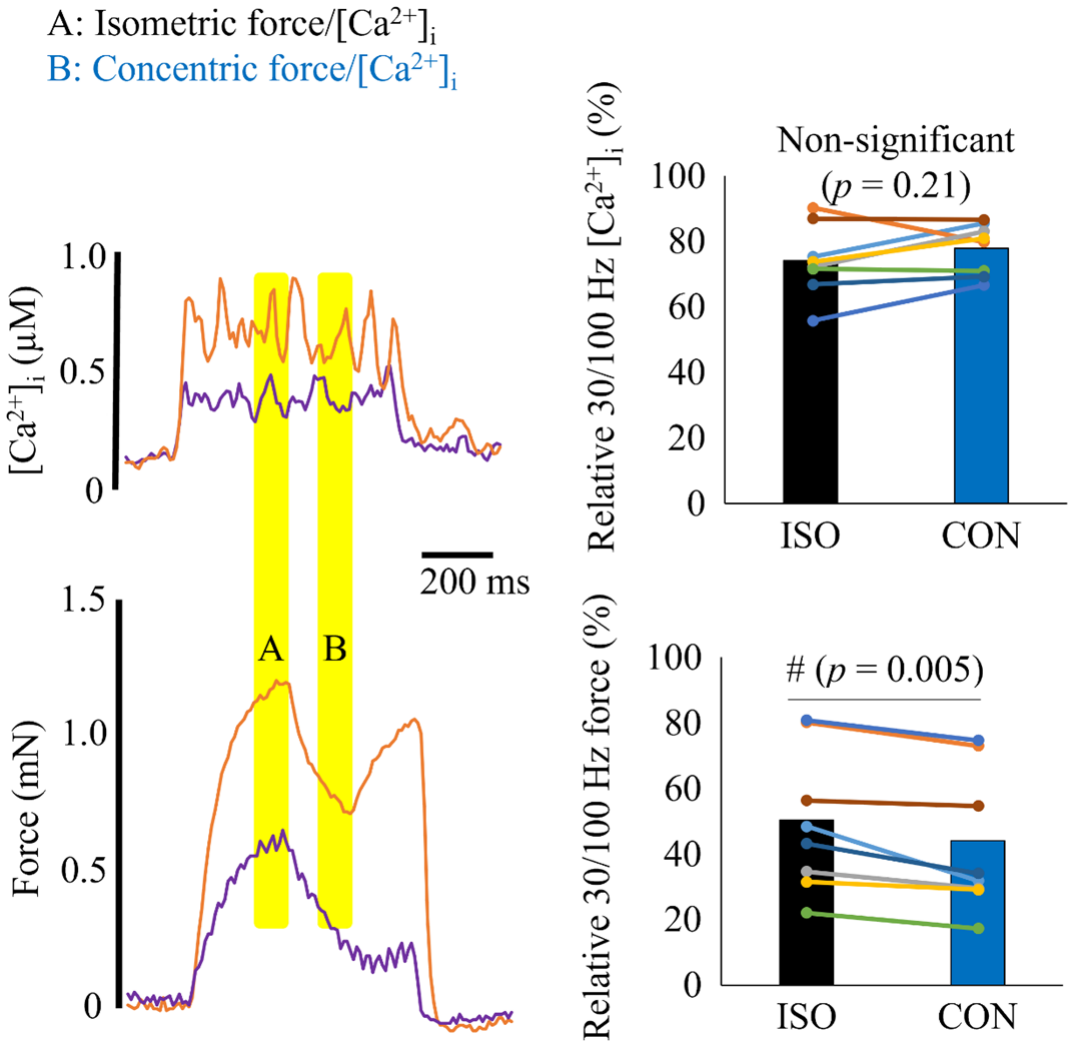
Typical [Ca^2+^]_i_ and force records obtained in 100 Hz (orange) and 30 Hz (purple) contractions during the concentric condition. Right part shows bars of mean values and individual values of the 30/100 Hz [Ca^2+^]_i_ and force ratios in 8 fibers. ISO, isometric phase (measured during yellow shading A); CON, concentric phase (measured during yellow shading B).

Figure 5 shows that the 30/100 Hz [Ca^2+^]_i_ ratio did not differ between concentric (78 ± 8%) and isometric (74 ± 11%) conditions (*p* = 0.21). In contrast, the 30/100 force ratio was significantly smaller in the concentric (44 ± 20%) than in the isometric (50 ± 20%) phase (*p* = 0.005).

**Figure 6:**
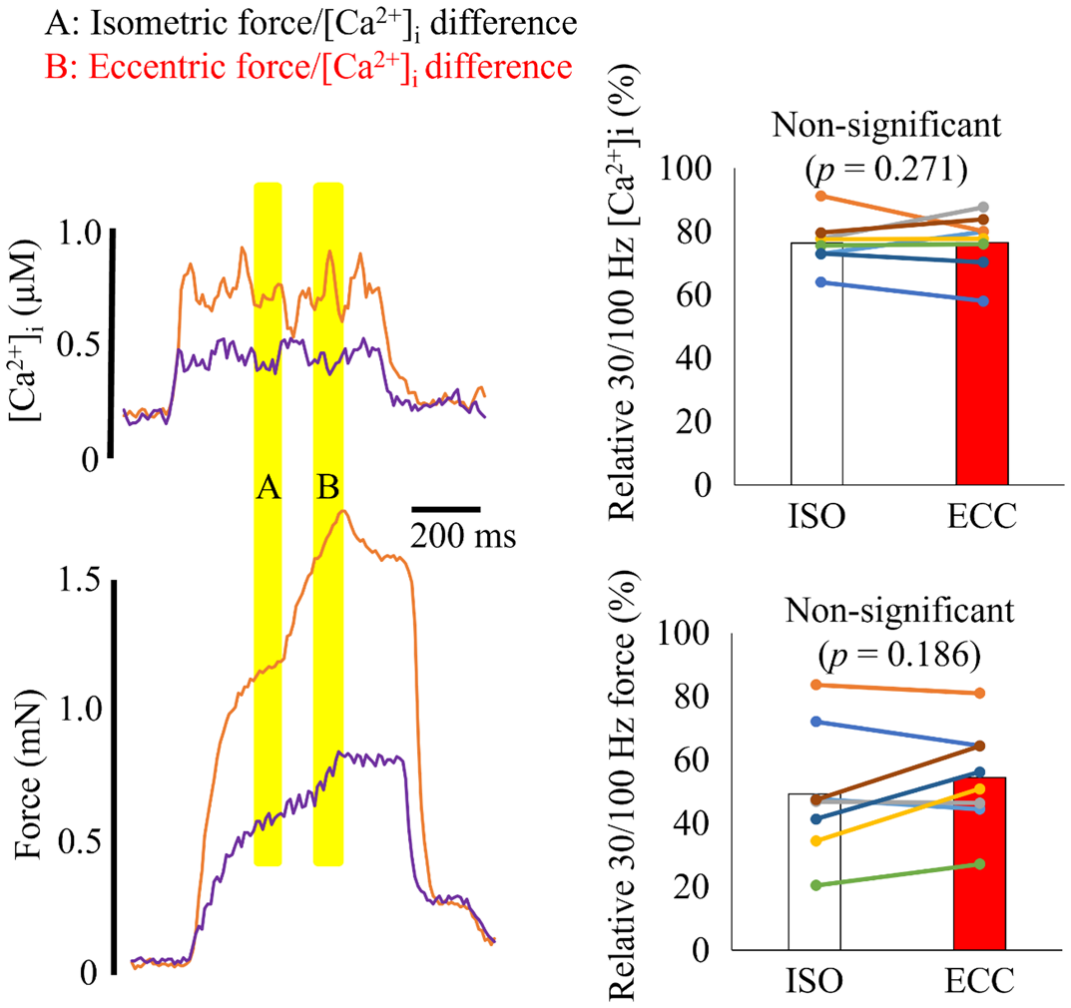
Typical [Ca^2+^]_i_ and force records obtained in 100 Hz (orange) and 30 Hz (purple) contractions during the eccentric condition. Right part shows bars of mean values and individual values of the 30/100 Hz [Ca^2+^]_i_ and force ratios in 8 fibers. ISO, isometric phase (measured during yellow shading A); ECC, eccentric phase (measured during yellow shading B).

Figure 6 shows that in the eccentric trial, there was no significant difference either in the 30/100 Hz [Ca^2+^]_i_ ratio (eccentric 77 ± 9%, isometric 76 ± 8%; *p* = 0.931) or the 30/100 Hz force ratio (eccentric 55 ± 16%, isometric 49 ± 20%; *p* = 0.186).

## Discussion

The main finding of this study is that tetanic [Ca^2+^]_i_ was similar during isometric, concentric and eccentric phases of dynamic contractions. To the best of our knowledge, tetanic [Ca^2+^]_i_ has not been measured during dynamic contractions in mammalian muscle. We found that the [Ca^2+^]_i_ concentration was the same during isometric, concentric, and eccentric contractions when the stimulation frequency was the same (Figs. 2, 3, 4). This result would be similar with the previous study reporting that the amount of [Ca^2+^]_i_ released was simply determined by the stimulation frequency not affected substantially by the muscle length^17^. However, our result differs from a previous study using intact single frog tibialis anterior muscle at 3 °C^18^. In this earlier study, once fibers were actively shortened, the Ca^2+^ indicator signal became slightly larger than that attained in the purely isometric contraction, and the increase was sustained even after the end of the active shortening (see Fig. 1 in Edman and Caputo^18^). One of the differences between this earlier study and our current study is that we used the ratiometric dye indo-1 to measure [Ca^2+^]_i_, while Edman and Caputo^18^ used the non-ratiometric Ca^2+^ dye fluo-3. Indo-1 has clear advantages over fluo-3 in that the emitted indo-1 fluorescence is measured at two wavelengths that are combined to make a ratio in contrast to the fluo-3 emitted fluorescence which is measured at only one wavelength and can thus be markedly affected by changes in fiber position and volume as the fiber length changes. This methodological difference can affect the contradicting results.

There are several studies examining the force-Ca^2+^ relationship and some conflicting results exists. For example, Vandenboom et al.^19^ performed similar experiments, and reported that Ca^2+^ concentration was increased during shortening. The possible differences which might explain this contradicted result between Vandenboom et al.^19^ and ours are that (1) amphibian (frog) versus mammalian (mouse), (2) low temperature (4 degrees) versus room temperature (22–24 degrees), and (3) very fast shortening (the force became zero) versus slower shortening (the substantial force was observed throughout the shortening phase). Especially, the shortening velocity should be critical. This is because Vandenboom et al.^19^ performed both very fast shortening and slow shortening conditions and reported that the Ca^2+^ concentration was increased only during very fast shortening (although Vandenboom et al.^19^ did not show any data about slow shortening condition). This idea is supported by the study^20^ reporting that Ca^2+^ concentration was not changed when relatively slow length changes were imposed during the isometric tetanus. Based on these results and our current results, it would be reasonable to assume that in the physiological (relatively slow) shortening velocities, Ca^2+^ concentration is not affected by length changes.

One of possible explanations for the different force-stimulation frequency, stimulation frequency-pCa relationship among contraction types is mechano-sensing of the thick filament (myosin ON–OFF transition). The previous study reported that the transition from the OFF state to the ON state was saturated about 50% of the maximal isometric force^21^. Therefore, the proportion of the ON state myosin heads would be saturated in both isometric and concentric contraction in the 100 Hz condition. On the other hand, the force was smaller in the 30 Hz condition and smaller in the concentric contraction than in the isometric contraction. This smaller concentric force (i.e., the smaller mechanical stress applied to the myosin filament) in the 30 Hz condition can make more myosin as the OFF state. In that situation, the force might be decreased more in the concentric conditions although the Ca^2+^ concentration is decreased equally in the concentric and isometric conditions. This point should be an interesting research topic in the field of muscle mechanics.

Although the behavior of cross-bridges during dynamic contraction remains elusive, Piazzesi et al.^22^ reported that when the shortening speed was increased, the force was decreased as expected, and this decreased force was explained by the decreased number of attached cross-bridges not the decreased force per attached cross-bridge (smaller strain of the attached cross-bridge). Based on this finding, more Ca^2+^ might be required in the concentric than in the isometric contractions to attach available myosin heads to the actin filament (completely open the myosin biding sites on the actin filament by Ca^2+^). This might be one of possibilities for the different force-stimulation frequency (Ca^2+^) relationship between concentric and isometric contractions.

We observed a larger force decrease during shortening in submaximal (30 Hz) than in near-maximal (100 Hz) contractions in the concentric contraction. A force reduction during active shortening due exclusively to compromised cross-bridge force-generating capacity would be expected to be of the same relative magnitude in submaximal and near-maximal contractions. On the other hand, a shortening-induced force reduction that also involves decreased Ca^2+^ activation of the thin filaments would be expected to be relatively larger in submaximal contractions, which take place at the steep part of the force-Ca^2+^ relationship, than at 100 Hz, which is close to maximum force^23^. Thus, the lower 30/100 Hz force ratio during active shortening than during isometric contraction is likely to reflect weakened cooperativity of strong cross-bridge attachments and facilitated troponin C Ca^2+^ binding, hence resulting in reduced thin filament activation^24^^,^^25^.

In contrast to the concentric contraction, the eccentric contraction shows the similar 30/100 force ratio. This result shows that force responses by changing the stimulation frequency (Ca^2+^) is similar between isometric and eccentric contractions.

One limitation of the present study is that the initial isometric part of contractions was produced at optimal length, and hence the concentric and eccentric phases took place at slightly below and above optimal length, respectively. Force-frequency and force-[Ca^2+^]_i_ relationships can be affected by muscle length^17^. However, the effect of muscle fiber length per se is likely to be small in the present study since the imposed length changes were small (only up to 5% of the muscle fiber length). Another limitation of the current study is that we used the FDB muscles that are mainly composed of fast twitch fibers^26^, and thus our findings might not be applicable to slow twitch fibers. Baylor and Hollingworth^27^ reported that the peak [Ca^2+^]_i_ attained during maximal intensity contractions was more than two times larger in fast twitch (extensor digitorum longus) than in slow twitch (soleus) mouse muscle fibers. This indicates that Ca^2+^ release and removal kinetics differ between fast and slow twitch fibers, which might affect the [Ca^2+^]_i_− and force-frequency relationships. Interestingly, a previous study on human knee extensor muscles (composed of both fast and slow twitch fibers, Johnson et al.^28^) of three individuals showed differences in the force-frequency relationships between isometric and concentric contractions that were similar to those observed in the present study^11^.

## Conclusions

Our results show that the force-frequency relationship differs between the isometric and concentric phases of contraction. Specifically, the force attained at the 30 Hz stimulation relative to the 100 Hz stimulation was smaller in the concentric than in the isometric contraction although the [Ca^2+^]_i_ was equally decreased between concentric and isometric contractions. We conclude that since the [Ca^2+^]_i_-frequency relationship was not different between contraction types, the reduced relative force during active shortening at low stimulation frequencies is caused by the decreased myofibrillar Ca^2+^ sensitivity.

### Supplementary Information

Supplementary Information.

## Data Availability

All data generated or analyzed during this study are included in this published article and its supplementary information files.
